# Chromo-Fluorogenic Detection of Soman and Its Simulant by Thiourea-Based Rhodamine Probe

**DOI:** 10.3390/molecules24050827

**Published:** 2019-02-26

**Authors:** Shengsong Li, Yongchao Zheng, Weiqiang Chen, Meiling Zheng, He Zheng, Zhe Zhang, Yan Cui, Jinyi Zhong, Chonglin Zhao

**Affiliations:** 1 Research Institute of Chemical Defense, Beijing 102205, China; dotasongge@163.com (S.L.); fhyjyzh@126.com (H.Z.); loopifer@163.com (Z.Z.); tracypiscescy@163.com (Y.C.); 2 State Key Laboratory of NBC Protection for Civilian, Beijing 102205, China; 3 Institute of Modern Physics, Chinese Academy of Sciences, Lanzhou 730000, China; chenwq7315@impcas.ac.cn; 4 Laboratory of Organic NanoPhotonics and CAS Key Laboratory of Bio-Inspired Materials and Interfacial Science, Technical Institute of Physics and Chemistry, Chinese Academy of Sciences, Beijing 100190, China; zhengmeiling@mail.ipc.ac.cn

**Keywords:** Nerve agents, Soman, Rhodamine B, Thiosemicarbazide, Chromo-fluorogenic probe, Detection

## Abstract

Here, we introduced a novel thiourea-based rhodamine compound as a chromo-fluorogenic indicator of nerve agent Soman and its simulant diethyl chlorophosphate (DCP). The synthesized probe N-(rhodamine B)-lactam-2-(4-cyanophenyl) thiourea (RB-CT), which has a rhodamine core linked by a cyanophenyl thiosemicarbazide group, enabled a rapidly and highly sensitive response to DCP with clear fluorescence and color changes. The detection limit was as low as 2 × 10^−6^ M. The sensing mechanism showed that opening of the spirolactam ring following the phosphorylation of thiosemicarbazides group formed a seven-membered heterocycle adduct, according to MS analysis and TD-DFT calculations. RB-CT exhibited high detecting selectivity for DCP, among other organophosphorus compounds. Moreover, two test kits were employed and successfully used to detect real nerve agent Soman in liquid and gas phase.

## 1. Introduction

**Scheme 1:**
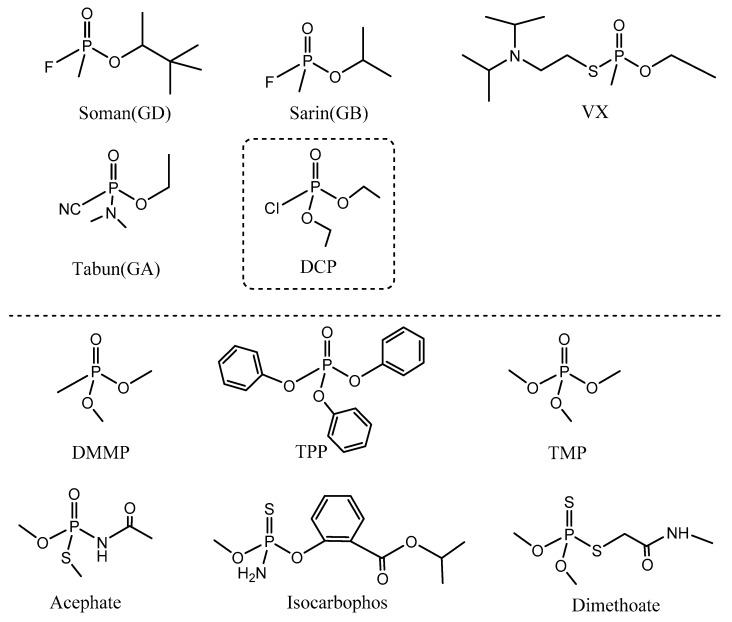
Chemical structures of nerve-agents Sarin, Soman, Tabun, and VX, their simulant DCP, and some organophosphorus compounds as potential interferences.

Nerve agents are a class of highly toxic organophosphates, mainly including Tabun (GA), Sarin (GB), Soman (GD), and VX [1] (Scheme 1). These compounds are extremely dangerous because they are able to enter human body through respiration or penetration and inhibit the activity of the acetylcholinesterase, then the accumulation of acetylcholine in the synapse causes neuromuscular paralysis and eventually death [2]. Due to the easy production, high toxicity, being colorless and odorless, and the possible use in terrorist attacks, the development of reliable and rapid detection systems for nerve agents is highly desirable [3]. In addition, rapidly discriminating the distribution of nerve agents in the contaminated areas can also provide effective guidance for subsequent decontamination operations and further safety confirmation.

Several methodologies have been employed for the detection of nerve agents, including enzymatic assays [4,5], interferometry [6], ion mobility spectroscopy [7], electrochemistry [8], micro-cantilevers [9,10], and photonic crystals [11]. Nevertheless, these protocols usually have some drawbacks, such as operation complexity, non-portability, difficulties in real-time monitoring, etc. As alternatives to these procedures, some new approaches have been explored involving molecularly imprinted polymers [12], nanoparticles [13], and chromo-fluorogenic probes [14,15,16,17,18,19,20,21,22]. Above all, chromo-fluorogenic probes have recently gained increasing interest due to their cost-effectiveness, simplicity, and “naked-eye” detection [23,24].

A typical chromo-fluorogenic probe is usually formed by two moieties: (1) a chromo-fluorogenic reporter group, which translates the binding event into the change of color and fluorescence, mainly containing rhodamine [22], fluorescein [15], boron dipyrromethene (BODIPY) [16,17], azo [14,20], and cyanine dye [18]; (2) a selective reactive group, which provides a reactive binding site for nucleophilic attack, mainly containing hydroxyl [15,25], oxime [19], and amino groups [26]. Recently, thiourea has been proved to be capable of reacting with nerve agents through hydrogen-bond interaction between N-H protons of thiourea and phosphonate oxygen or hydrolyzed products [27,28]. It has been also proven that the reaction of thiosemicarbazide group can induce opening of the spirolactam ring in rhodamine B accompanied with color change and enhanced fluorescence [29]. Thus, the thiosemicarbazide group is expected to become a reactive group bonding with rhodamine B core for chromo-fluorogenic detection of nerve agents and simulants.

**Scheme 2:**
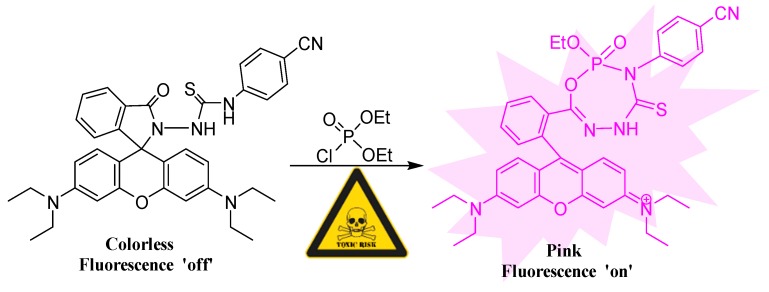
Proposed mechanism of RB-CT with DCP.

Herein, a rhodamine B based probe N-(rhodamine B)-lactam-2-(4-cyanophenyl) thiourea (RB-CT) was designed and synthesized, and as shown in Scheme 2, the electron-withdrawing cyanophenyl group in the molecule could promote the intramolecular charge transfer (ICT) process to enhance activity of ring-opening reaction and strengthen the change of color. Spectra analysis and mechanism study confirmed the probe was capable of the rapid detection of diethyl chlorophosphate (DCP, a simulant of the Soman) with remarkable change of color and fluorescence through the phosphorylation of the thiosemicarbazide group. Finally, the probe was also successfully used in the detection of GD in both liquid and gas phase.

## 2. Results and Discussion

### 2.1. Spectroscopic Properties

**Figure 1:**
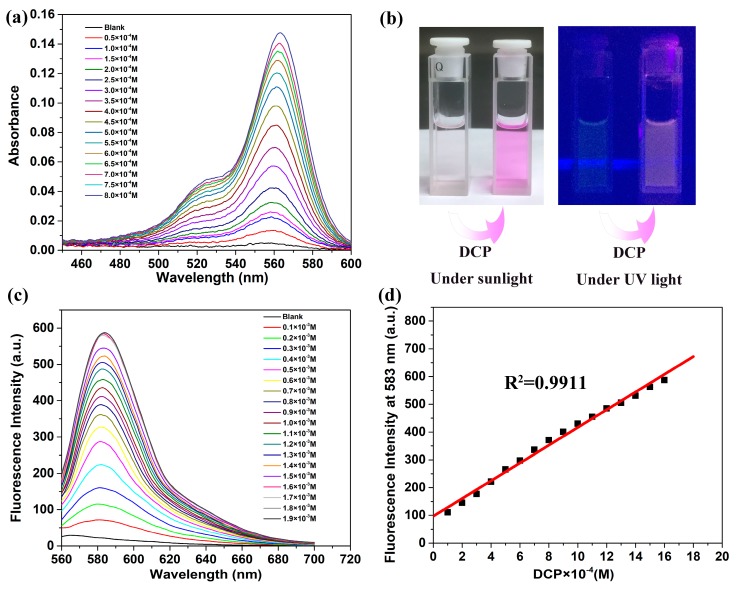
(**a**) Absorption spectra of RB-CT (1.0 × 10^−5^ M) upon the addition of DCP (0.5 × 10^−4^–8.0 × 10^−4^ M) in CH_3_CN (3% Et_3_N). (**b**) The images of the RB-CT solution before and after the addition of DCP under sun light (left) and UV light (right). (**c**) Fluorescence spectra of RB-CT (1.0 × 10^−6^ M) at λ_ex_ = 540 nm, upon the addition of DCP (0.1 × 10^−3^–1.9 × 10^−3^ M) in CH_3_CN (3% Et_3_N). (**d**) Plot of emission intensity of RB-CT at 583 nm.

To demonstrate the response of RB-CT to nerve agents, absorption and fluorescence spectra were first studied. DCP was used as low-toxic simulant because of its similar chemical structure and reactivity with nerve agents (Tabun, Sarin and Soman). Et_3_N (3 Vt%) was added in the CH_3_CN solution of RB-CT to avoid the interference of proton. As shown in Figure 1a, an obvious absorption band at 560 nm was observed after the addition of DCP in the RB-CT solution. The change in color from colorless to pink implied the spirolactam ring-opening reaction of RB-CT caused by DCP (Figure 1b). The thiosemicarbazide group of RB-CT is likely to react with DCP, resulting in a remarkable enhancement of absorption intensity. The result highlights that RB-CT is of high potential as a naked-eye probe for the detection of nerve agents.

Figure 1c presented the fluorescence spectra of RB-CT with increasing concentration of DCP; a new fluorescent band at 583 nm appears and shows remarkable enhancement. The spectral changes provided further evidence for the opening of the spirolactam ring, according to the changes in absorption spectra. Moreover, the fluorescence intensity of RB-CT showed a good linear relationship at the concentration of DCP in the range of 0.1 × 10^−3^–1.9 × 10^−3^ M (Figure 1d).

The limit of detection (LOD) was determined from the fluorescence spectral data, using the equation K × S_b1_/S, where K = 3, S_b1_ is the standard deviation of blank measurements and S is the slope of the calibration curve. The limit of detection was found to be 2 × 10^−6^ M, indicating high sensitivity to detect DCP by RB-CT. The sensing ability for the naked eye was evaluated by immobilizing RB-CT on silica plates. After the plates were dipped in the CH_3_CN solution of DCP (2–2000 ppm), clear enhancement of the fluorescence was observed by the naked eye in the range of 20 and 2000 ppm (Figure S1), which demonstrates the LOD for the naked eye is as low as the value calculated.

### 2.2. Reaction Kinetics Study

**Figure 2:**
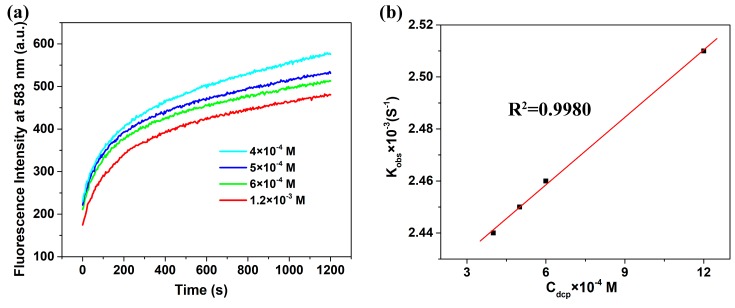
(**a**) Kinetic profiles of the fluorescence intensity at 583 nm of RB-CT ((1.0 × 10^−6^ M, CH_3_CN, 3% Et_3_N) after the addition of 4.0 × 10^−4^ M, 5.0 × 10^−4^ M, 6.0 × 10^−4^ M, and 1.2 × 10^−3^ M DCP. (**b**)The correlation between k_obs_ and the concentration of DCP.

To investigate the reaction kinetics of RB-CT and DCP, time-dependent fluorescence change was performed under different DCP concentrations at room temperature. As shown in Figure 2a, the reaction was almost complete and the fluorescence intensity was saturated within 1200 s in all cases. The kinetic constants were further examined following pseudo-first-order kinetic rate law as the clear linear relationships between the change of fluorescence intensity and reaction time (Figure S2). The observed rate constants k_obs_, half-life time t_1/2_, and the constant rates (k) were summarized in Table 1. The average half-life time was about 280 s, less than 5 min. The short half-life times are the basis for the rapid detection of nerve agents.

### 2.3. Mechanism Study

**Figure 3:**
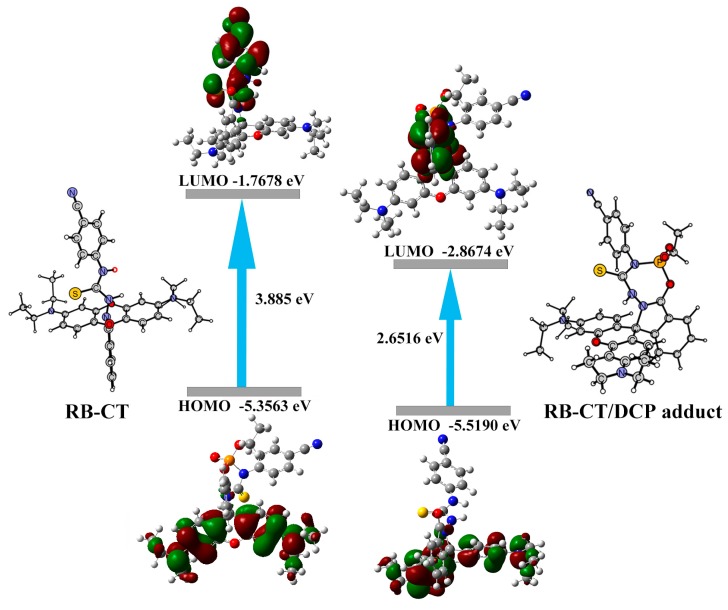
Energy optimized structures, HOMO-LUMO energy levels, and interfacial plots of the orbitals of RB-CT and RB-CT/DCP adduct.

The absorption and fluorescence changes can be attributed to the opening of spirolactam ring via nucleophilic reaction between RB-CT and DCP. An ESI analysis was employed to investigate the possible products and a major peak at 708.3223 was observed, which indicated the phosphorylation of RB-CT (Figure S3). The new singlet observed (δ 0.32) in the ^31^P NMR spectrum of DCP after addition of RB-CT gives further evidence for the occurrence of phosphorylation reaction (Figure S4). As the formation of a heterocycle in RB-CT/DCP adduct is a reasonable mechanism [30,31], DFT and TD-DFT calculations were used to confirm the structure of the adduct through optimizing the geometries formed by nucleophilic attack with different nitrogen sites in the thiosemicarbazide group. The proposed mechanism was presented in Scheme 2; a seven-membered heterocycle was proved to be a favorable adduct structure owing to its relatively lower energy, which also agreed well with the mass spectrometry result (Figure S5). The optimized structures of RB-CT and RB-CT/DCP adducts were shown in Figure 3. The HOMO–LUMO energy gap (2.651 eV) of the adduct is less than that of RB-CT (3.885 eV) and the electron densities in LUMO are more concentrated in the thiourea group after the reaction. The calculated main contributing electronic transitions for S_0_→S_1_ energy state are HOMO→LUMO (2.14 eV/578 nm) and HOMO-1→LUMO+1 (2.34 eV/530 nm), which is consistent with the absorption band at 560 nm obtained experimentally. The considerable difference in the energy and electronic transition can shed light on the changes of absorption spectra and color.

### 2.4. Interferents

**Figure 4:**
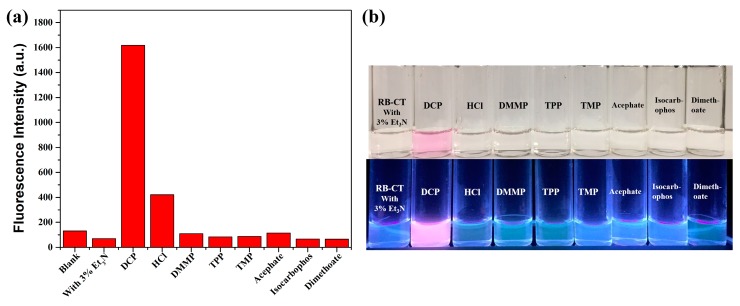
(**a**) Relative fluorescence intensity of RB-CT (1 × 10^−5^ M, CH_3_CN, 3% Et_3_N) at 583 nm after the addition of different organophosphorus compounds (1.0 × 10^−3^ M). Blank is the CH_3_CN solution of RB-CT without Et_3_N. (**b**) Relative color change of RB-CT after the addition of different organophosphorus compounds under sunlight and UV light.

As organophosphorus pesticides often act as interferences to render false positives during the detection of nerve agents, several conventional organophosphorus compounds were chosen as target species to study the selectivity of RB-CT. As shown in Figure 4, the interferences did not cause any obvious color and fluorescence changes in DCP, which indicated the selective detection of DCP can be achieved by RB-CT, among other conventional organophosphorus compounds. It is noted that although proton can induce the spirolactam ring opening of the rhodamine molecule [32], RB-CT exhibited little changes of color and fluorescence intensity in the presence of hydrochloric acid. Thus, the false-positive caused by proton can be effectively avoided under the detection condition with the addition of 3% Et_3_N, since the suitable detection pH range of the probe is 7.0 to 10.0 (Figure S6).

### 2.5. Practical Application toward Real Nerve Agent

**Figure 5:**
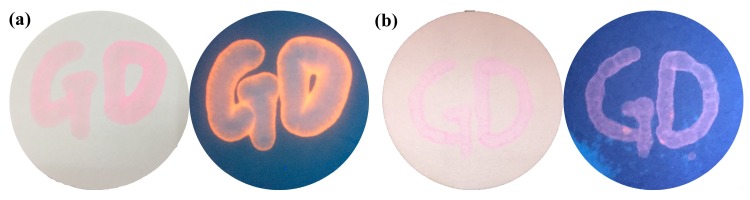
(**a**) Color change of filter paper treated with RB-CT solution (1.0 × 10^−4^ M in CH_3_CN, 3% Et_3_N) and GD in liquid phase under sunlight (left) and UV light (right). (**b**) Color change of filter paper treated with RB-CT solution (1.0 × 10^−4^ M in CH_3_CN, 3% Et_3_N) and exposed to GD in gas phase under sunlight (left) and UV light (right).

To confirm in situ and rapid sensing ability of RB-CT toward real nerve agents, we have investigated the response characteristics of the probe to GD in both liquid and gas phase. Clear color changes under sunlight and fluorescence enhancement irradiated by UV lamp were all observed by the naked eye immediately in liquid and within 10 min in gas phase (40 ppm GD introduced in a flask as an aerosol) (as shown in Figure 5). The remarkable visual differences did not fade or quench after 24 h, which indicated the irreversible cyclization had occurred [33]. The above results illustrate the potential application of RB-CT in the rapid and facile naked-eye detection of nerve agents in both liquid and vapor phases.

## 3. Materials and Methods

### 3.1. Materials

All of the solvents were obtained from Beijing Chemical Reagent Company and used without further purification. Rhodamine B, 4-cyanophenyl isothiocyanate and anhydrous magnesium sulfate (MgSO_4_) were purchased from Aladdin In. Co. (Los Angeles, CA, USA). Diethyl chlorophosphate (DCP) was purchased from Sigma Co. (Louis, MO, USA). Nerve agent GD (the purity is 80 %) was provided by the Research Institute of Chemical Defense of China.

### 3.2. Measurements

The ESI mass analysis was performed using an Q Exactive HF-X Hybrid Quadrupole-Orbitrap Mass Spectrometer (Thermo Scientific, Waltham, MA, USA). The ^1^H NMR spectra were recorded on AV 400 spectrometer (Bruker, Karlsruhe, Germany). Chemical shifts were expressed in parts per millions (δ) downfield from the internal standard tetramethyl silane and were reported as s (singlet), d (doublet), bs (broad singlet), t (triplet), and m (multiplet). Absorbance and fluorescence spectra were recorded at room temperature with a Hitachi U-3900 UV-Visible spectrophotometer and F-4500 fluorescence spectrophotometer, respectively, using a fluorescence cell of 10 mm path. The excitation wavelength was set to 540 nm (slit width 5 nm), and emission was monitored from 560–700 nm (slit width 5 nm). Column chromatography was conducted over silica gel (mesh 100–200).

### 3.3. Synthetic Procedures

**Scheme 3:**
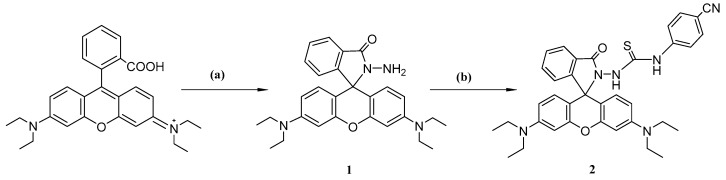
Synthesis route of RB-TU. Reagents and conditions: (a) CH_3_OH, hydrazine hydrate, 80 °C, 6 h; (**b**) DMF, 4-cyanophenyl isothiocyanate, r.t, 12 h.

As shown in Scheme 3, compound 1 was synthesized using the reported procedure [34]. Compound 2 was synthesized in a facile way.

**Synthesis of compound 1:** In a 100 mL flask, 1 mL hydrazine hydrate was drop-wise added to a solution of rhodamine B (1.67 mmol, 800 mg) in anhydrous CH_3_OH (30 mL), the mixed solution was then stirred and heated to 80 ℃, and refluxed for 6 h. Pure water (30 mL) was added and the solvent was extracted with EtOAc (3 × 60 mL), after the organic phase was dried with anhydrous MgSO_4_ and evaporated, orange solid compound 1 (356 mg, 48.8% yield) was obtained, and used without further purification.

The ^1^H NMR (400 MHz, DMSO-d6) δ 7.82 (d, *J* = 8.3 Hz, 1H), 7.58-7.48 (m, 2H), 7.04 (d, *J* = 8.0 Hz, 1H), 6.41 (d, *J* = 17.1 Hz, 6H), 4.32 (s, 2H), 3.36 (d, *J* = 7.0 Hz, 8H), 1.14 (t, *J* = 6.9 Hz, 12H) (Figure S7).

**Synthesis of compound 2(RB-TU probe):** To a solution of compound 1 (0.22 mmol, 100 mg) in 1.5 mL DMF, a solution of 4-cyanophenyl isothiocyanate (0.31 mmol,50 mg) in 1.5 mL DMF was added, the reaction mixture was stirred for 12 h at room temperature. After the solution evaporated, the residue was purified by flash chromatography (EtOAc: hexane = 1:10) to afford RB-TU probe (36 mg, 26.6% yield).

The ^1^H NMR (400 MHz, Chloroform-d) δ 8.01 (d, *J* = 7.6 Hz, 1H), 7.72 - 7.57 (m, 3H), 7.44 (d, *J* = 8.4 Hz, 2H), 7.34 (d, *J* = 8.5 Hz, 2H), 7.29 (d, *J* = 7.6 Hz, 1H), 6.97 (s, 1H), 6.47 (d, *J* = 8.8 Hz, 2H), 6.42 (d, *J* = 2.5 Hz, 2H), 6.29 (d, *J* = 2.6 Hz, 1H), 6.27 (d, *J* = 2.6 Hz, 1H), 3.32 (qd, *J* = 7.2, 2.5 Hz, 8H), 1.15 (t, *J* = 7.0 Hz, 12H) (Figure S8).

HR-MS (C_36_H_36_N_6_O_2_S): Calcd.: 616.2620; Found: 617.2681(9.9 ppm) (Figure S9).

### 3.4. Reaction Kinetics Study

The fluorescence spectra of RB-CT (1.0 × 10^−6^ M) were recorded after the addition of DCP in the concentrations of 4.0 × 10^−4^ M, 5.0 × 10^−4^ M, 6.0 × 10^−4^ M, and 1.2 × 10^−3^ M over a 0–1200 s incubation period at room temperature. The observed rate constants k_obs_ and corresponding half-life time t_1/2_ were determined according to Equations (1) and (2).

ln[(F_max_-F_t_)/F_max_] = −k_obs_t (1)

t_1/2_ = ln2/k_obs_ (2)

where F_max_ and F_t_ are maximum fluorescence intensity obtained after the reaction was completed and the fluorescence intensities at 583 nm at reaction time t.

### 3.5. Computational Methods

Quantum chemical calculations were carried out by Gaussian 09 package [35]. Geometry was optimized using density functional theory (DFT) functional B3LYP [36,37,38] and 6–31G (d) basis set [39,40]. Time-dependent density functional theory (TD-DFT) calculation [41] was also performed at the same level of theory. Considering the effects of the environment on the calculated energies, solvation effects were considered throughout geometry optimizations via the self-consistent reaction field (SCRF) and conductor-like polarizable continuum model (CPCM) method [42].

### 3.6. Interferents

Selectivity and specificity tests were performed with different organophosphorus compounds (OPs), i.e., dimethyl methylphosphonate (DMMP), triphenyl phosphate (TPP), trimethyl phosphate (TMP), acephate, isocarbophos, and dimethoate (Scheme 1). To 2 mL reagent solution of the probe molecule (1.0 × 10^−5^ M) in CH_3_CN containing 3% Et_3_N, 20 μL stock solution of OPs (1.0 × 10^−1^ M) was added. The solutions were incubated at room temperature for 5 min and the fluorescence spectra was recorded.

### 3.7. The Preparation of Test Kits of Real Nerve Agents in Liquid and Gas Phase

The test kits toward GD in liquid and gas phase were produced by the following steps. For liquid phase, a waterman filter paper was soaked in the CH_3_CN solution of RB-CT (1.0 × 10^−4^ M, 3% Et_3_N), after the solvent was air-dried, a “GD” logo was patterned by GD. For gas phase, a solution of RB-CT in CH_3_CN (1.0 × 10^−4^ M, 3% Et_3_N) was sprayed onto a filter paper to spell out “GD” first, after the solvent was air-dried, the paper was exposed to GD vapor in a flask (40 ppm introduced as an aerosol) for 10 mins. The LCt_50_ and LD_50_ of Soman are estimated to be 100 mg min/m^3^ and 300 mg/individual, respectively [43].

Safety Note: Only highly qualified and experienced personnel should work with CWAs employed here, as described in the experimental part below. Due to the high volatility and toxicity of the CWAs, all of the experiments in this part were carried out inside a safety fume hood and operated under efficient ventilation systems. To avoid any risk of CWA inhalation, respiratory protection of involved personnel was provided by protective breathing masks equipped with combined NBC filters.

## 4. Conclusions

In conclusion, we have designed and synthesized a novel probe N-(rhodamine B)-lactam-2-(4-cyanophenyl) thiourea for chromogenic and fluorogenic detection of nerve agents. The probe undergoes an irreversible opening-ring reaction following the form of a seven-membered heterocycle adduct in the presence of DCP, accompanied by the obvious color change from colorless to pink, which has been confirmed by MS analysis and TD-DFT calculations. The response is that instantaneously half-life time is about 280 s at room temperature and the detection limit was found to be 2 × 10^−6^ M. Furthermore, we have demonstrated that the probe is applicable for rapid and facile in situ detection of GD with the naked eye in both liquid and vapor phase. 

## Figures and Tables

**Table 1:** Observed reaction rates (k_obs_), half-life time (t_1/2_), and the constant rates (k) for the reaction of RB-CT ((1.0 × 10^−6^ M, CH_3_CN, 3% Et_3_N) and DCP with different concentrations.

The Concentrations of DCP	4 × 10^−4^	5 × 10^−4^	6 × 10^−4^	1.2 × 10^−3^
**K_obs_ (s^−1^)**	0.00244	0.00245	0.00246	0.00251
**t_1/2_ (s)**	284	283	282	276
**k (M^−2^ S^−1^)**	8.645 × 10^−6^			

## Supplementary Materials

The following are available online. Figure S1: The limit of detection for naked eye. Figure S2: Calibration plots by using fluorescence intensity of RB-CT (1 µM) as a function of reaction time t in different DCP concentrations. Figure S3: ESI-MS spectrum of RB-CT/DCP adduct. Figure S4: ^31^P NMR spectra of DCP and RB-CT with DCP in CD_3_CN. Figure S5: HOMO-LUMO energy levels of TD-DFT optimized geometries of probe and predicted RB-CT/DCP adducts. Figure S6: The plot of pH versus fluorescence intensity of free RB-CT and RB-CT+DCP. Figure S7: ^1^H NMR Spectrum of Compound 1. Figures S8 and S9: ^1^H NMR and ESI-MS Spectra of RB-CT.
