# Supplementary material for: Chromo-Fluorogenic Detection of Soman and Its Simulant by Thiourea-Based Rhodamine Probe
Source: Molecules. 2019 Feb 26;24(5):827. doi: 10.3390/molecules24050827 (PMC6429212; doi:10.3390/molecules24050827)
Supplement: Supplementary file 1 [file molecules-24-00827-s001.pdf]

Supporting information

Chromo-fluorogenic Detection of Soman and Its Simulant by Thiourea-based Rhodamine Probe

Shengsong Li [1,†], Yongchao Zheng [1,4,†,], Weiqiang Chen [2], Meiling Zheng [3], He Zheng [1,4], Zhe Zhang [1], Yan Cui [1,4], Jinyi Zhong [1,4,*], Chonglin Zhao [1,4,*]*

1 Research Institute of Chemical Defense, Beijing 102205, P. R. China; dotasongge@163.com (S.S.L.); fhyjyzh@126.com (H.Z.); loopifer@163.com (Z.Z.); tracypiscescy@163.com (Y.C.)
2 Institute of Modern Physics, Chinese Academy of Sciences, Lanzhou 730000, P. R. China; chenwq7315@impcas.ac.cn (W.Q.C.)
3 Laboratory of Organic NanoPhotonics and CAS Key Laboratory of Bio-Inspired Materials and Interfacial Science, Technical Institute of Physics and Chemistry, Chinese Academy of Sciences, Beijing 100190, P. R. China; zhengmeiling@mail.ipc.ac.cn (M.L.Z.)
4 State Key Laboratory of NBC Protection for Civilian, Beijing 102205, P. R. China;
* Correspondence: zhengycfh@163.com (Y.C.Z.); linfzjy@163.com (J.Y.Z.); zhaochonglin@126.com (C.L.Z.); Tel.: +86-010-66758321(C.L.Z.)
† These authors have contributed equally to this work.

Contents

1. The limit of detection for naked eye………………………………………………………... **S1**
2. Calibration plots by using fluorescence intensity of RB-CT (1 μM) as a function of reaction time t in different DCP concentrations…………………………………………………………... **S2**
3. ESI-MS spectrum of RB-CT/DCP adduct…………………………………………………… **S3**
4. ^{31}P NMR spectra of DCP and RB-CT with DCP in CD_3CN……………………………. …. **S4**
5. HOMO-LUMO energy levels of TD-DFT optimized geometries of probe and predicted RB-CT/DCP adducts……………………………………………………………………………. **S5**
6. pH study……………………………………………………………………………………... **S6**
7. ^{1}H NMR Spectrum of Compound 1………………………………………………………….. **S7**
8. ^{1}H NMR and ESI-MS Spectra of RB-CT………………………………………. ……...**S8**

1. The limit of detection for naked eye

Processed silica plates were soaked in RB-CT solution (1.0×10^{-5} M, CH_3CN,3% Et_3N) for 5 min and air-dried at room temperature for 2 h for further test. Then the plates were dipped in the CH_3CN solution of DCP (2-2000 ppm) and a UV lamp was used to investigate the change of fluorescence. A strong enhancement of the fluorescence was observed by the naked eye in the range of 20 and 2000 ppm while no fluorescence was observable before they were dipped, which demonstrates the LOD for naked eye is as low as the value calculated.

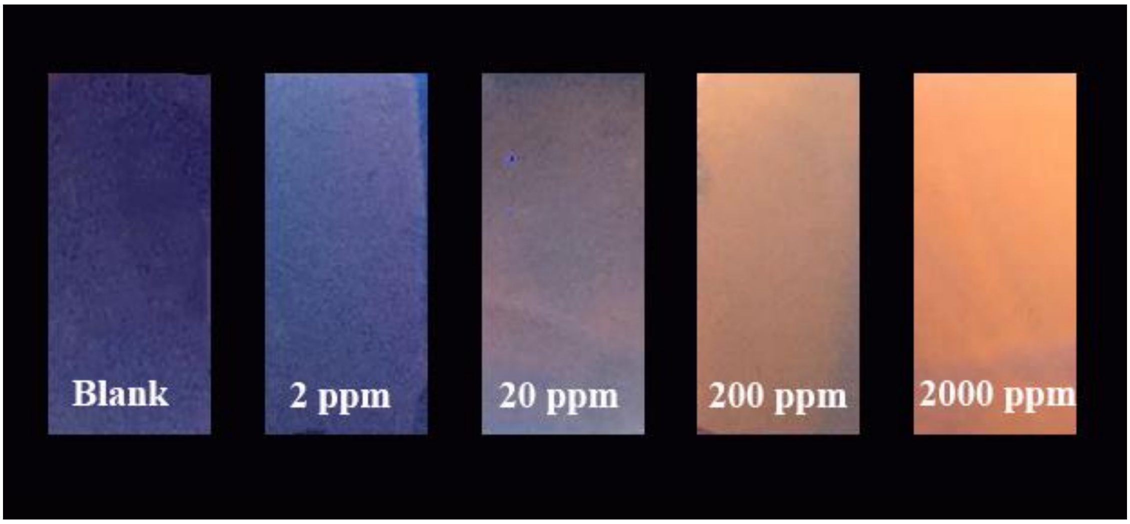

Figure S1. Images of RB-CT (1.0×10^{-5} M, CH_3CN,3% Et_3N) doped silica plates after dipping in the CH_3CN solution of DCP (0, 2, 20, 200 and 2000 ppm).

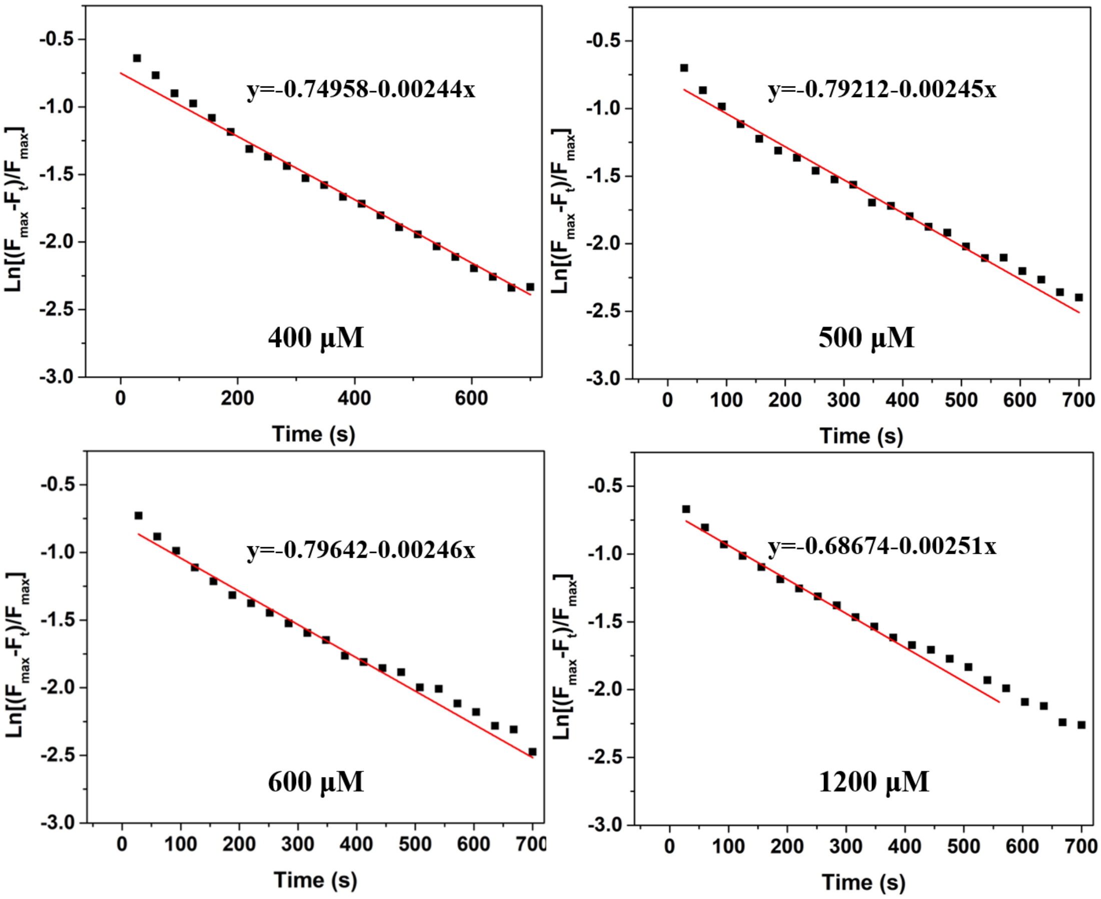

Figure S2. Calibration plots by using fluorescence intensity of RB-CT (1 µM) as a function of time t in different DCP concentrations (400 µM, 500 µM, 600 µM and 1200 µM).

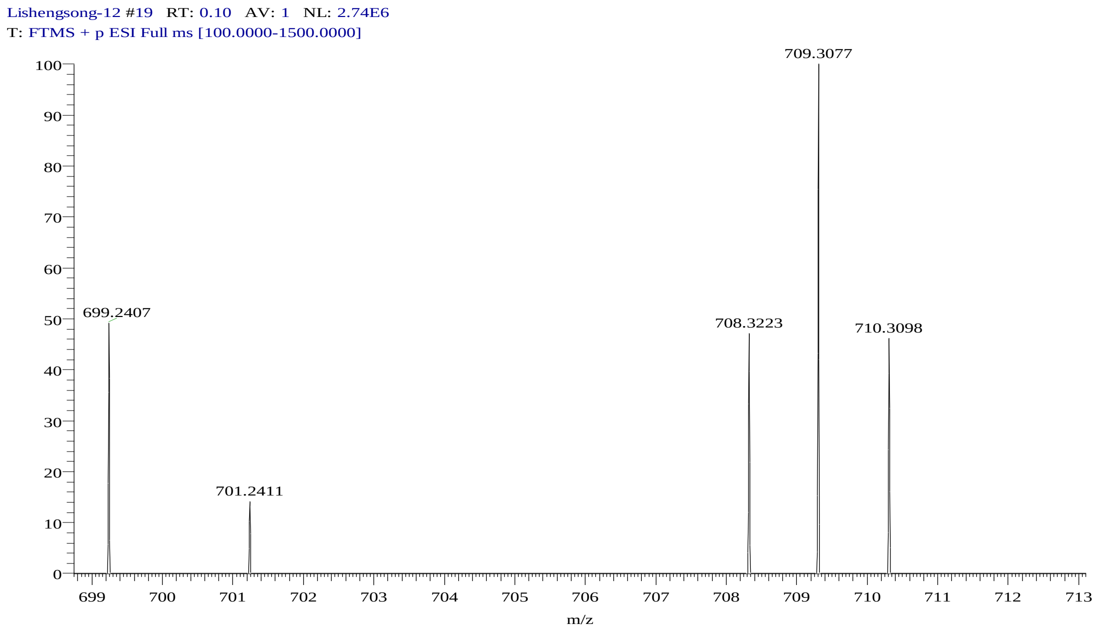

Figure S3. ESI-MS spectrum of **RB-CT/DCP** adduct

An ESI analysis of RB-CT in CH_3CN solution (3% Et_3N) after the addition of DCP (5 equiv.) showed a major peak situated at 708.3223, which is consistent with theoretical molecular weight of RB-CT/DCP adduct.

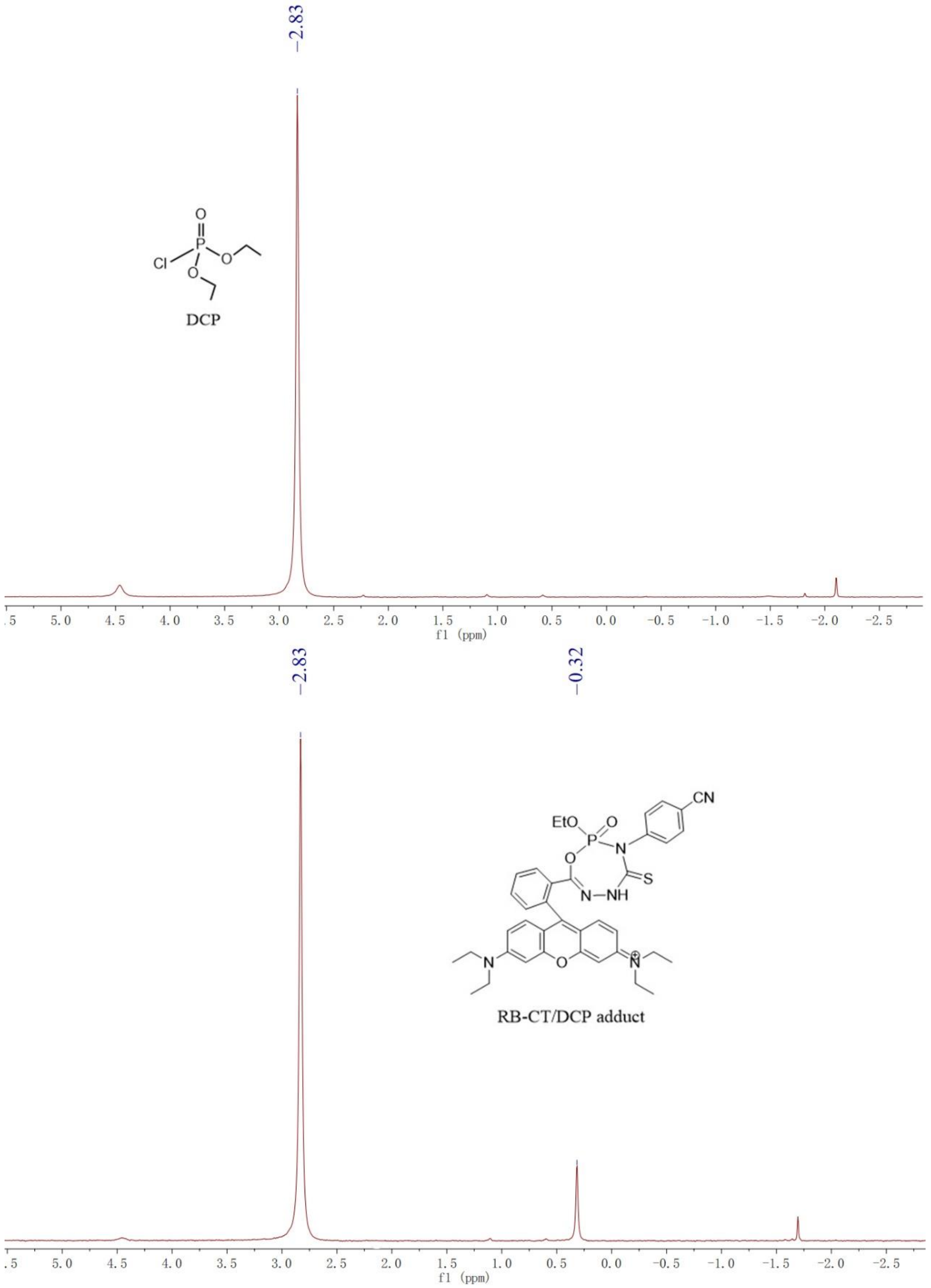

Figure S4. ${}^{31}P$ NMR spectra of DCP (a) and RB-CT with DCP (b) in CD_3CN.
A new singlet at 0.32 was found in the ${}^{31}P$ NMR spectrum of DCP (10 mM) after the addition of RB-CT (2 mM).

Molecular structures	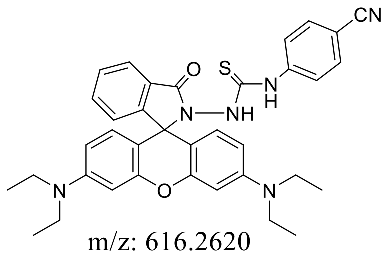 m/z: 616.2620	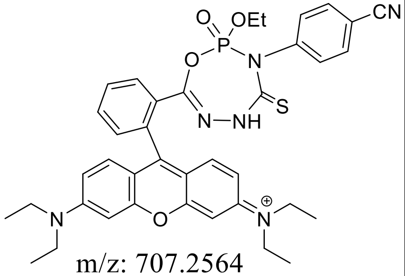 m/z: 707.2564	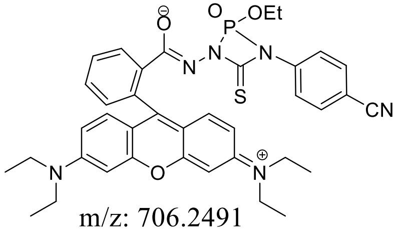 m/z: 706.2491	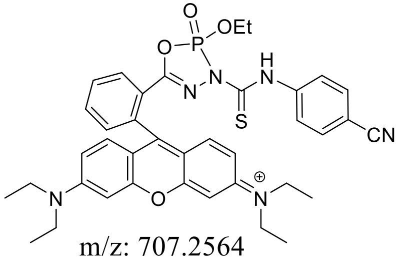 m/z: 707.2564
Optimized structures	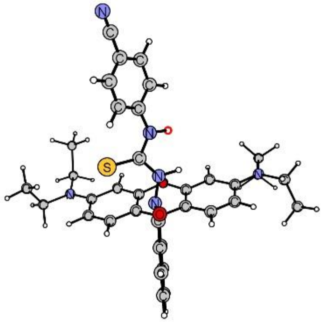	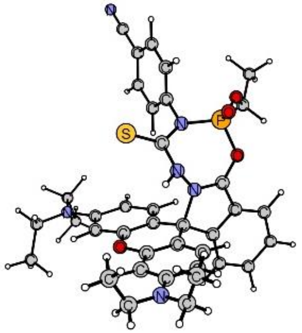	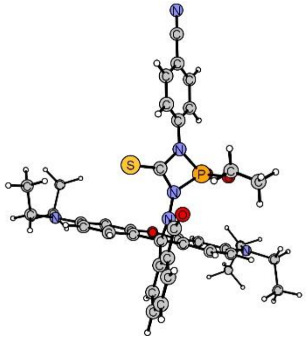	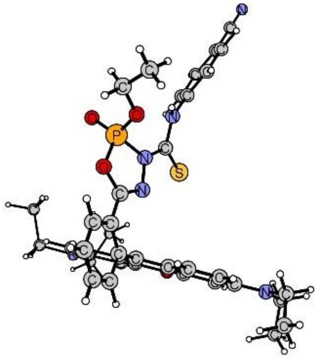
E (a.u.)	-2270.833161	-2841.149727	-2841.185657	-2840.713821
HOMO (a.u.)	-0.19685	-0.20283	-0.21051	-0.19142
LUMO (a.u.)	-0.06497	-0.10538	-0.10956	-0.06771
ΔE(a.u.)	0.13188	0.09745	0.10095	0.12371
ΔE(ev)	3.5884548	2.6516145	2.7468495	3.3661491

Figure S5. HOMO-LUMO energy levels of TD-DFT optimized geometries of probe and predicted RB-CT/DCP adducts.

5. pH studies

RB-CT solution (1.0×10^{-5} M, CH_3CN/H_2O 1:1,v/v) was titrated with aqueous solutions of 1 M Hydrochloric acid or 1 M sodium hydroxide to adjust pH into 4-11 range monitored by pH meter, followed with the addition of 100 equivalent DCP.

RB-CT exhibited weak fluorescence between pH 7.0 and 10.0, and obvious fluorescence enhancement was observed after the addition of DCP because of the opening of the spirolactam ring. The result suggests that RB-CT is appropriate to be a DCP probe in comparatively wide pH range (7-10).

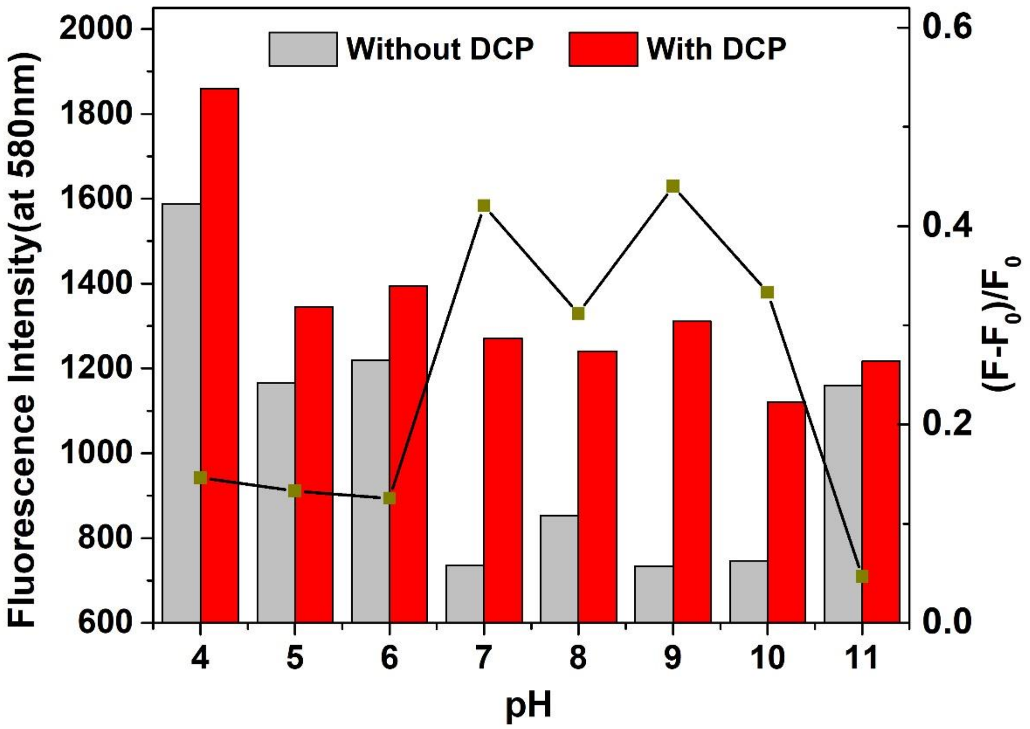

Figure S6. The plot of pH versus fluorescence intensity of free RB-CT (gray color) and RB-CT+DCP (red color).

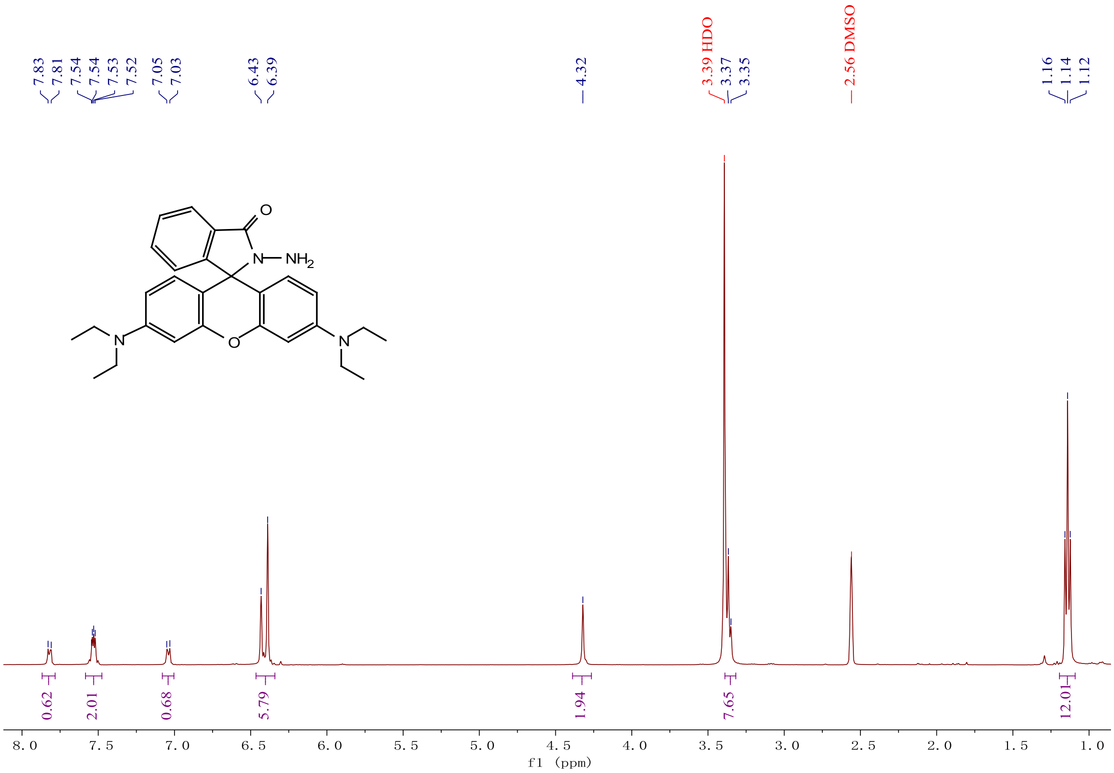

Figure S7. ^{1}H NMR Spectrum of Compound 1 (400 MHz, DMSO-d_6) δ 7.82 (d, J = 8.3 Hz, 1H), 7.58-7.48 (m, 2H), 7.04 (d, J = 8.0 Hz, 1H), 6.41 (d, J = 17.1 Hz, 6H), 4.32 (s, 2H), 3.36 (d, J = 7.0 Hz, 8H), 1.14 (t, J = 6.9 Hz, 12H).

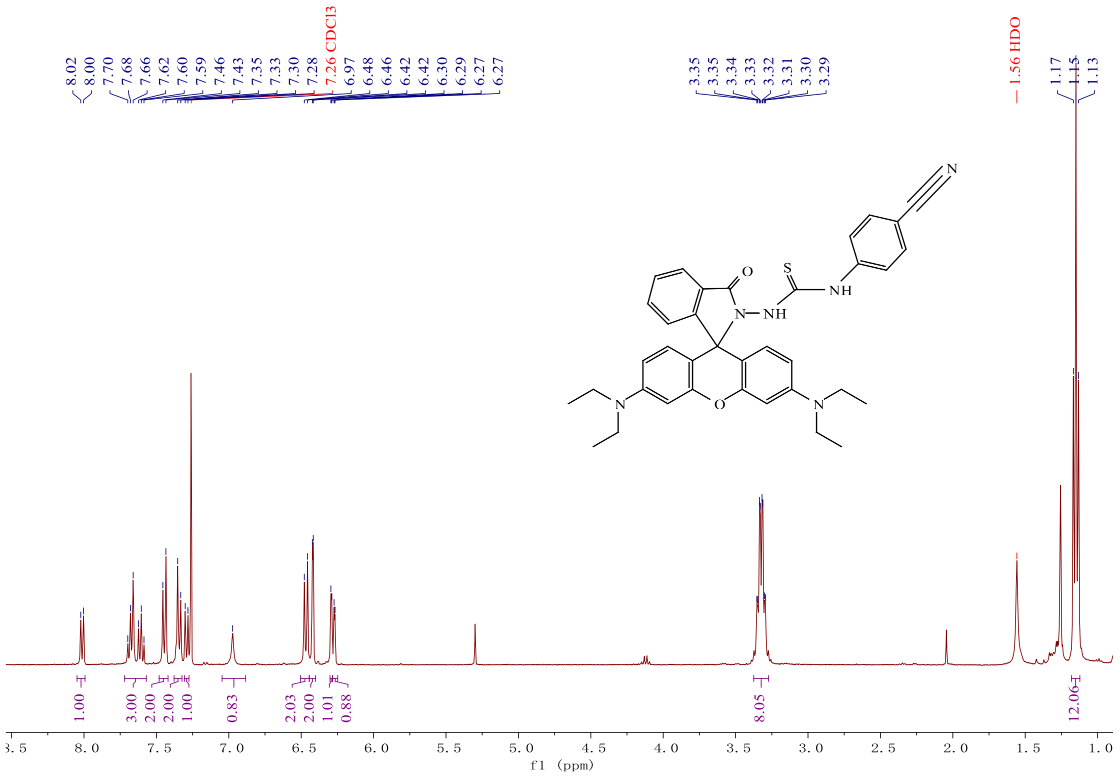

Figure S8. ^{1}H NMR Spectrum of RB-CT (400 MHz, Chloroform-*d*) δ 8.01 (d, *J* = 7.6 Hz, 1H), 7.72 - 7.57 (m, 3H), 7.44 (d, *J* = 8.4 Hz, 2H), 7.34 (d, *J* = 8.5 Hz, 2H), 7.29 (d, *J* = 7.6 Hz, 1H), 6.97 (s, 1H), 6.47 (d, *J* = 8.8 Hz, 2H), 6.42 (d, *J* = 2.5 Hz, 2H), 6.29 (d, *J* = 2.6 Hz, 1H), 6.27 (d, *J* = 2.6 Hz, 1H), 3.32 (qd, *J* = 7.2, 2.5 Hz, 8H), 1.15 (t, *J* = 7.0 Hz, 12H).

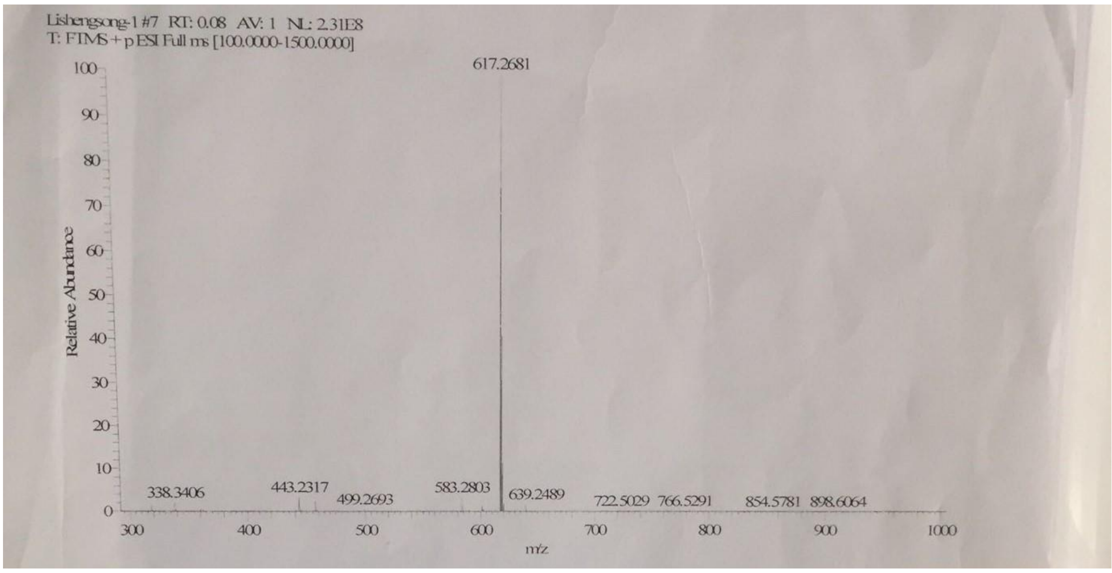

Figure S9. ESI-MS spectrum of RB-CT.

m/z Calcd.: for $C_{36}H_{36}N_6O_2S$ 616.2620; Found: 617.2681 (9.9 ppm).
